# Continence Recovery After Radical Prostatectomy: Personalized Rehabilitation and Predictors of Treatment Outcome

**DOI:** 10.3390/diagnostics15222881

**Published:** 2025-11-13

**Authors:** Małgorzata Terek-Derszniak, Danuta Gąsior-Perczak, Małgorzata Biskup, Tomasz Skowronek, Mariusz Nowak, Justyna Falana, Jarosław Jaskulski, Mateusz Obarzanowski, Stanislaw Gozdz, Pawel Macek

**Affiliations:** 1 Department of Rehabilitation, Holycross Cancer Centre, Artwinskiego 3, 25-734 Kielce, Poland; malgorzatater@onkol.kielce.pl (M.T.-D.); malgorzatabi@onkol.kielce.pl (M.B.); tomaszsk@onkol.kielce.pl (T.S.); mariuszno@onkol.kielce.pl (M.N.); 2 Collegium Medicum, Jan Kochanowski University in Kielce, Zeromskiego 5, 25-369 Kielce, Poland; danutagp@onkol.kielce.pl (D.G.-P.); jaroslawja@onkol.kielce.pl (J.J.); mateuszob@onkol.kielce.pl (M.O.); stanislawgozdz1@gmail.com (S.G.); 3 Endocrinology Clinic, Holycross Cancer Centre, 25-734 Kielce, Poland; 4 Oncology Clinic, Holycross Cancer Centre, Artwinskiego 3, 25-734 Kielce, Poland; justynafa@onkol.kielce.pl; 5 Department of Urology, Holycross Cancer Centre, Artwinskiego 3, 25-734 Kielce, Poland; 6 Scientific Research, Epidemiology and R&D Centre, Holycross Cancer Centre, Artwinskiego 3, 25-734 Kielce, Poland

**Keywords:** urinary incontinence, radical prostatectomy, pelvic floor muscle training, continence recovery, robot-assisted radical prostatectomy, pad test, physiotherapy, predictive factors

## Abstract

**Background/Objectives**: Urinary incontinence (UI) remains a common and distressing complication following radical prostatectomy (RP). This prospective observational study aimed to assess the effectiveness of structured pelvic floor rehabilitation and to identify clinical and surgical predictors of continence recovery. **Methods**: A total of 182 patients undergoing RP received standardized physiotherapist-guided pelvic floor muscle training (PFMT), including supervised sessions before and after surgery, as well as individualized home exercise programs. UI severity was evaluated using a 1 h pad test and a four-level UI stage classification at three time points. The primary outcomes were changes in UI stage and the achievement of full continence, defined as a pad test result ≤2 g. **Results**: Following three rehabilitation sessions, 80.2% of patients regained full continence. Preoperative PFMT (β = −1.27, *p* = 0.0061) and shorter time to rehabilitation (β = −0.04, *p* = 0.0026) were associated with greater improvement in continence outcomes. Patients treated with robot-assisted RP showed a higher probability of continence recovery compared to those undergoing laparoscopic RP, particularly in the presence of moderate to severe baseline incontinence. Higher baseline urinary leakage significantly decreased the odds of treatment success (β = −0.01, *p* = 0.0001). ISUP grade and extraprostatic extension were not independently associated with outcomes. **Conclusions**: Despite the absence of a control group, this study demonstrates the effectiveness of structured and personalized pelvic floor rehabilitation in improving post-RP continence. Early initiation and preoperative training should be prioritized to optimize recovery in routine clinical practice.

## 1. Introduction

Urinary incontinence (UI) is one of the most common and burdensome complications following radical prostatectomy (RP) [1,2,3,4]. The incidence of UI varies depending on several factors, including the surgical technique used, the definition of incontinence applied, and the duration of follow-up. According to the literature, up to 80% of patients experience stress urinary incontinence immediately after surgery [5]. Although symptoms are often transient, a substantial proportion of patients, approximately 20–40%, continue to struggle with UI one year after robot-assisted radical prostatectomy (RARP) [6]. Notably, these rates are lower compared to open prostatectomy, highlighting the influence of surgical approach on incontinence risk [7,8]. Postoperative UI is multi factorial, resulting primarily from sphincteric damage, inadequate urethral support from the pelvic floor muscles, and, in some cases, neurological impairment. The condition has a significant impact on patients’ quality of life, leading to social withdrawal, reduced professional activity, and a heightened risk of depressive symptoms [9]. Given these consequences, the management of UI should be comprehensive and multidisciplinary, encompassing preventive, therapeutic, and educational interventions targeting patients, caregivers, and healthcare providers. Physiotherapy plays a key role in conservative treatment of UI. The American Urological Association (AUA) recommends pelvic floor muscle training (PFMT) as the first-line therapy both before and after RP [10]. When combined with biofeedback, electrical stimulation, and manual therapy, PFMT aims to improve muscular function and stimulate the nervous system. There is growing emphasis on initiating PFMT in the preoperative period. Studies suggest that learning proper pelvic floor activation before surgery may enhance the effectiveness of therapy and accelerate continence recovery [11]. Preoperative preparation allows patients to train under comfortable conditions, free from postoperative pain or sensory disturbances. The efficacy of physiotherapy, both before and after surgery, has been confirmed in numerous systematic reviews and meta-analyses [2,12,13,14,15]. However, the literature still lacks conclusive evidence regarding the optimal timing, intensity, and patient-related predictors of therapy success. Personalized rehabilitation, which tailors the type and intensity of exercises to each patient’s clinical and surgical profile, may lead to improved outcomes and more efficient use of healthcare resources [11].

The aim of this study was twofold: (1) to evaluate the effectiveness of a standardized pelvic floor muscle rehabilitation program in improving urinary continence following radical prostatectomy, and (2) to identify clinical and surgical predictors of treatment response and the number of rehabilitation sessions required to achieve continence.

## 2. Materials and Methods

### 2.1. Study Design Consideration

This was a prospective observational study conducted in a clinical setting where pelvic floor muscle training (PFMT) has been a standard component of post-prostatectomy care for several years. For ethical and practical reasons, it was not feasible to implement a control group without rehabilitation, as withholding physiotherapy would have deprived patients of an established element of routine care. The study was therefore designed to assess treatment effectiveness by analyzing continence outcomes and their predictors over time, within a cohort receiving standardized pelvic floor rehabilitation. While causal inference is limited in the absence of a randomized control group, multivariable modeling was employed to identify independent associations and reduce confounding.

The study cohort consisted of 182 male patients scheduled for radical prostatectomy (RP) due to localized prostate cancer. Among them, 106 underwent laparoscopic radical prostatectomy (LRP) and 76 had robot-assisted radical prostatectomy (RARP). The average patient age was 66.1 years (SD 6.5). All participants were referred to a physiotherapist approximately one month before surgery.

Eligibility criteria included: male sex, age ≥ 18 years, diagnosis of localized prostate cancer, and qualification for RP. Participants had to be free of any neurological or urological disorders that could influence urinary continence and were required to provide written informed consent. Patients were excluded if they had contraindications to physiotherapy, declined participation, or had incomplete clinical records.

#### 2.1.1. Rehabilitation Protocol

The rehabilitation process was structured into four distinct stages. The initial stage (Stage 0) was implemented approximately one month prior to surgery. A total of 146 patients completed three physiotherapy sessions under professional supervision, while the remaining 36 were unable to attend due to individual circumstances.

During this preparatory stage, patients were trained in locating, activating, and controlling pelvic floor muscles. Instruction was supported by surface electromyography (sEMG; Noraxon Ultium, Noraxon U.S.A. Inc., Scottsdale, AZ, USA) using intra-anal probes and 50-mm surface electrodes (INTCO, Shandong INTCO Medical Technology Co., Ltd., Zibo, Shandong Province, China), alongside ultrasound imaging with the Medison SonoAce PICO device (Medison Co./Samsung Medison, Seoul, Republic of Korea).

#### 2.1.2. Detailed First Session Procedure

During the first postoperative session, patients engaged in exercises involving deliberate pelvic floor muscle contractions performed in various body positions, including prone, sitting, and standing. These included isolated pelvic floor muscle exercises, quick-release contractions, and exercises synchronized with the breathing cycle. Patients were instructed to perform pelvic floor and breathing exercises four times daily, completing ten repetitions per set in different body positions, with a particular emphasis on standing and sitting. In addition, each patient received a home exercise program involving lower limb and pelvic girdle exercises and was advised to walk for at least 30 min daily. This rehabilitation approach was personalized in nature. Each session was guided by a specialized physiotherapist who adapted the training plan according to the patient’s functional status, UI severity, and individual progress. Adjustments were made based on clinical feedback, pad test results, and observed performance during exercises. Personalized components included tailored instruction, correction of improper muscle activation patterns (e.g., use of abdominal or gluteal muscles), and real-time feedback using sEMG and ultrasound. This individualized strategy allowed the physiotherapist to optimize the rehabilitation plan dynamically, ensuring that patients received support appropriate to their continence recovery trajectory. After the catheter was removed, patients continued with the same pelvic floor exercises. Rehabilitation Stage 1 began approximately one month later. During the first postoperative session, the physiotherapist reassessed pelvic floor function and performed a standardized 1 h pad test (PT) using Seni Man Level 4 Extra Plus pads to measure urinary leakage. The pad was weighed before placement under the penis, positioned inside the patient’s underwear. The assessment protocol included a sequence of activities designed to provoke urinary leakage: drinking 0.5 L of water over 15 min while seated; walking for 30 min (including stair use); 10 repetitions of standing up from a chair; lifting a weight from the floor 5 times; jogging in place for 1 min; coughing 10 times; and washing hands under running water for 1 min.

Upon completion of the pad test protocol, which included a standardized sequence of activities designed to provoke urinary leakage, the pad was sealed in a plastic bag and weighed again using a medical scale (WLC6/F1/R; precision 0.1 g). Urinary incontinence (UI) was defined as a urine loss greater than 2 g. Based on the 1 h pad test results, UI was categorized into the following stages: Stage I: 2–10 g, Stage II: 11–50 g, Stage III: ≥50 g. Although Stage 0 was defined as urine loss ≤2 g, the mean value of 3.2 ± 15.7 g reflects the presence of a few outlier measurements with minimal leakage, while the median value was 0 g. This minor discrepancy does not affect the categorical classification of continence stages, which was based on the predefined cut-offs of 2, 10, and 50 g.

In addition to the pad test, the number of pads and other absorbent hygiene products used (including incontinence pads and adult diapers) was also documented.

Patients continued pelvic floor muscle and breathing exercises during five scheduled rehabilitation sessions. Stages II and III of the physiotherapy program took place three and six months post-catheter removal, respectively. At each time point, the physiotherapist reassessed pelvic floor muscle function and repeated the pad test.

Individuals with urine loss exceeding 50 g during testing and/or signs of neurological sensory impairment received supplementary neuromuscular electrostimulation, provided that their serum PSA levels remained within normal limits.

The following clinical variables—previously recognized in the literature as predictors of postoperative incontinence—were included in the analysis: age, body mass index (BMI), baseline incontinence stage, time from surgery to rehabilitation onset, and baseline pad test result [16,17,18,19].

The study was approved by the Bioethics Committee of Collegium Medicum, Jan Kochanowski University, Kielce, Poland (approval no. 34/2018, approval date: 28 May 2018).

#### 2.1.3. Adherence and Dose

Patient adherence to the rehabilitation program was monitored throughout the study. The structured pelvic floor muscle training (PFMT) protocol consisted of supervised sessions and a complementary home-based exercise program. Each participant attended a median of 6 supervised sessions (range: 4–8) led by a physiotherapist specializing in pelvic floor rehabilitation. Attendance was recorded for all visits, and 95% of patients completed at least 80% of the scheduled sessions.

Adherence to the home exercise program was assessed using patient diaries and physiotherapist notes collected during follow-up visits. Based on these reports, approximately 70% of participants performed home exercises at least five times per week as instructed.

Neuromuscular electrostimulation was applied in patients with minimal voluntary pelvic floor contraction at baseline (*n* = 42, 23%). The stimulation parameters were standardized: frequency 35 Hz, pulse width 250 μs, session duration 15 min, performed in combination with voluntary contractions.

A per-protocol sensitivity analysis restricted to participants who completed ≥80% of supervised sessions produced results consistent with the primary intention-to-treat analysis, confirming the robustness of findings.

#### 2.1.4. Statistical Analyses

The statistical strategy was aligned with the primary aim of the study: to assess the effectiveness of pelvic floor rehabilitation and to identify factors influencing continence outcomes and time to recovery. Both objective and clinical measures were analyzed over time, and appropriate regression models were applied to identify significant predictors of rehabilitation success.

Statistical analyses were performed using R software (version 4.4.1). All tests were two-sided, and a *p*-value of <0.05 was considered statistically significant. Continuous variables were analyzed using the Kruskal–Wallis test (for comparisons involving more than two groups), followed by Mann–Whitney U tests with Bonferroni correction for post hoc analyses. Categorical variables were compared using the chi-squared test, while McNemar’s test was applied for paired categorical data. Results were reported as means with standard deviations (SD) for continuous variables and as counts with percentages (*n*, %) for categorical variables. Baseline incontinence status was assessed using both the pad test result and the UI stage. To evaluate factors associated with baseline UI severity, linear regression and ordinal logistic regression models were employed. Rehabilitation effectiveness was assessed based on two outcomes: (1) change in UI stage between Examination 1 and Examination 3 (ΔUI stage), and (2) reduction in urinary leakage as measured by the pad test. Full continence was defined as a pad test result ≤2 g. To identify predictors of continence restoration after rehabilitation, logistic regression models were used. To assess predictors of time to continence recovery (i.e., the number of rehabilitation visits required to achieve a pad test result ≤2 g), ordinal logistic regression was applied. All multivariable models included clinical and pathological variables such as age, BMI, type of surgery, time to rehabilitation, preoperative PSA level, ISUP grade, extra prostatic extension (EPE), seminal vesicle invasion (SVI), and baseline pad test result. Model assumptions were verified prior to interpretation: for ordinal models, the proportional odds assumption was checked, and multicollinearity was assessed using variance inflation factors (VIF). Analyses were conducted on complete cases, as missing data were minimal and not assumed to be missing at random. We fitted a proportional-odds ordinal logistic regression with UI stage coded as an ordered outcome (0 < 1 < 2 < 3; higher values indicate more severe incontinence). Odds ratios (OR) and 95% confidence intervals (CI) were obtained by exponentiating model coefficients. OR < 1 indicates lower odds of being in a higher (i.e., more severe) UI stage, whereas OR > 1 indicates higher odds. Reference categories were ISUP 1, EPE = 0, and LRP (for surgery type).

## 3. Results

A total of 182 patients who underwent radical prostatectomy were included in the study to assess urinary incontinence and the effectiveness of pelvic floor rehabilitation (Table 1). The mean age was 66.1 years (SD 6.5), and the mean BMI was 28.2 kg/m^2^ (SD 3.6). The average preoperative PSA level was 9.2 ng/mL. The majority of patients received preoperative rehabilitation, and the mean time from surgery to the start of rehabilitation was 36.1 days. At baseline (Examination 1), approximately two-thirds of patients presented with urinary incontinence, most commonly classified as stage 2 or 3. The mean pad test result was 43.9 g, which improved significantly during rehabilitation, with a mean reduction of 36.0 g.

Surgery was performed using either laparoscopic (LRP) or robot-assisted (RARP) techniques, with RARP accounting for 41.8% of procedures. Histopathological analysis showed that most patients had pT2-stage tumors and ISUP grade 3 or 4. Adverse pathological features, such as extraprostatic extension (EPE) and seminal vesicle invasion (SVI), were less frequently observed.

### 3.1. Baseline Characteristics of Urinary Incontinence

#### 3.1.1. Differences in Incontinence Severity by UI Stage at Baseline

At the start of rehabilitation, pad test (PT) values differed significantly between groups stratified by UI stage at baseline. The Kruskal–Wallis test confirmed a statistically significant overall difference (*p* < 0.001), and post hoc pairwise comparisons using the Mann–Whitney U test with Bonferroni correction revealed significant differences between all UI stages except between Stage 2 and Stage 3. Pad test values increased with UI severity: UI = 0 (3.2 ± 15.7 g), UI = 1 (4.3 ± 1.7 g), UI = 2 (30.1 ± 12.4 g), UI = 3 (130.8 ± 83.1 g).

#### 3.1.2. Predictors of Baseline UI Stage—Ordinal Logistic Regression

**Figure 1:**
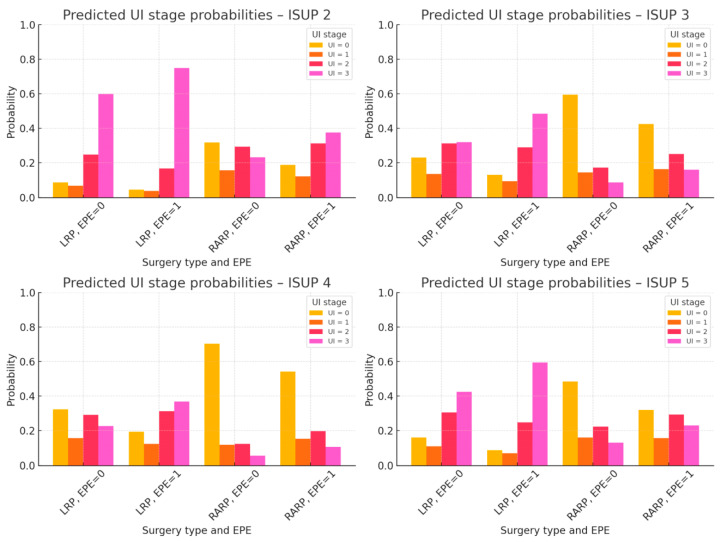
Predicted Probabilities of UI Stage (0–3) by ISUP Grade, Type of Surgery, and EPE Status. Caption: Predicted probabilities of each UI stage (0–3) are shown for combinations of ISUP grade (2–5), surgery type (LRP vs. RARP), and presence of extraprostatic extension (EPE), assuming average patient age. RARP is associated with lower probabilities of more severe urinary incontinence compared with LRP. Presence of EPE increases the probability of more severe UI stages. ISUP grades 2–4 are associated with lower probabilities of severe UI compared with ISUP 1, whereas ISUP 5 does not differ significantly. Abbreviations: UI, urinary incontinence; ISUP, International Society of Urological Pathology; LRP, laparoscopic radical prostatectomy; RARP, robot-assisted radical prostatectomy; EPE, extraprostatic extension.

To identify factors associated with baseline UI stage, an ordinal logistic regression model with a logit link function was applied (Table A1). The model included nine variables potentially related to incontinence severity: age, BMI, time to rehabilitation, preoperative rehabilitation, type of surgery, preoperative PSA level, presence of extraprostatic extension (EPE), seminal vesicle invasion (SVI), and ISUP grade. The analysis showed that RARP was significantly associated with a lower UI stage at baseline (see Table A1 and Figure 1). Preoperative rehabilitation demonstrated a trend toward significance. In the multivariable model, presence of extraprostatic extension (EPE 1 vs. EPE 0) was associated with higher odds of being in a more severe baseline UI stage (OR 2.39, 95% CI 1.11–5.10; *p* = 0.0266). Higher ISUP grades (2–4) showed lower odds of severe UI compared with ISUP 1 (ISUP 2: OR 0.24, 95% CI 0.08–0.74; *p* = 0.0135; ISUP 3: OR 0.34, 95% CI 0.12–1.01; *p* = 0.0516; ISUP 4: OR 0.21, 95% CI 0.07–0.66; *p* = 0.0076), whereas ISUP 5 was not significant. Other variables, including age, BMI, time to rehabilitation, PSA level and SVI, were not significantly associated with baseline UI severity (*p* > 0.05). Odds ratios and 95% confidence intervals are reported in Table A1.

Figure 1 shows the adjusted predicted probabilities of each UI stage (0–3) according to ISUP grade, type of surgery (RARP vs. LRP), and EPE status, assuming average age. RARP was associated with lower probabilities of severe UI stages compared with LRP. Presence of EPE was associated with higher probabilities of severe UI. ISUP grades 2–4 showed lower probabilities of severe UI than ISUP 1, whereas ISUP 5 did not differ significantly.

### 3.2. Effects of Rehabilitation and Factors Associated with Improvement

#### 3.2.1. Change in Continence Status over Time

**Figure 2:**
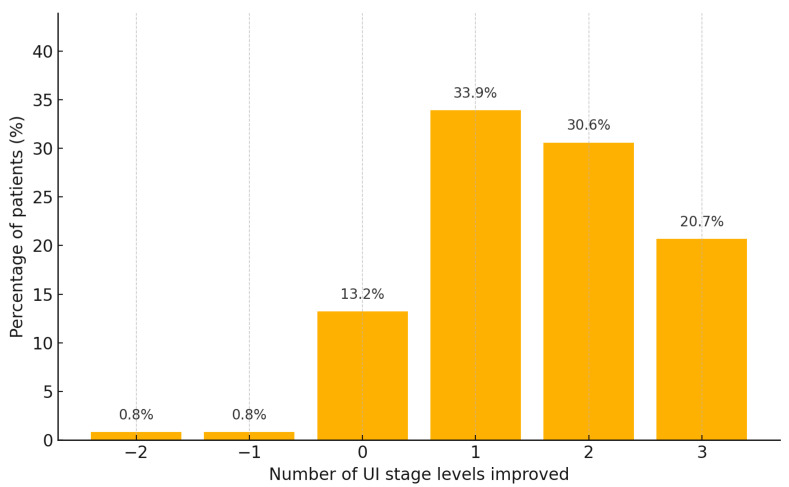
Distribution of improvement in UI stage between baseline and final assessment. The chart shows the distribution of patients according to the number of UI stage levels improved between Examination 1 and Examination 3. Only patients with a pad test result > 2 g at either time point were included in the analysis. Note: The most common improvement was by one UI stage (33.9%), followed by improvements of two (30.6%) and three stages (20.7%). Very few patients showed no change (13.2%) or deterioration (1.6%). Abbreviations: UI.

The proportion of patients achieving full continence, defined as a pad test result ≤ 2 g, increased progressively over the course of rehabilitation (Figure 2). After the first rehabilitation session, 34.1% of patients achieved full continence; this increased to 54.4% after the second session and to 80.2% following the third. These differences were statistically significant according to Mc Nemar’s test (*p* < 0.001). Among patients with urinary incontinence at baseline (pad test > 2 g, *n* = 120), most participants improved by one UI stage (33.9%), 30.6% improved by two stages, and 20.7% by three stages. Thirteen percent (13.2%) showed no change, while 1.6% experienced a worsening of symptoms.

#### 3.2.2. Factors Associated with Improvement in UI Stage

To identify factors associated with improvement in urinary incontinence, defined as the change in UI stage between Examination 1 and Examination 3, an ordinal logistic regression model with a logit link function was applied (Table 2). The analysis was restricted to patients with a pad test result > 2 g at baseline (*n* = 120), thereby excluding individuals without clinically relevant incontinence at the start of observation. In the multivariable model, preoperative rehabilitation was significantly associated with a higher likelihood of UI stage improvement (β = −1.27, *p* = 0.0061). Additionally, a shorter time to rehabilitation initiation significantly increased the likelihood of greater pelvic floor function recovery (β = −0.04, *p* = 0.0026). Other factors, including age, BMI, PSA level, type of surgery, local tumor advancement (EPE, SVI), and tumor grade (ISUP), were not significantly associated with improvement in UI stage. Although RARP was not significantly associated with the magnitude of improvement in UI stage, it was a significant predictor of lower baseline incontinence severity. This indicates that patients treated with RARP started rehabilitation from a more favorable continence status rather than experiencing a greater treatment effect.

#### 3.2.3. Achievement of Continence at the End of Therapy (Examination 3)

To identify predictors of full continence, defined as a pad test result ≤2 g at the third evaluation, a logistic regression analysis was conducted, including the baseline pad test result and other clinical and demographic variables (Table 3). In the multivariable model, a higher baseline pad test result was significantly associated with lower odds of achieving full continence (β = −0.01, *p* = 0.0001). Additionally, undergoing RARP was significantly associated with a greater likelihood of continence at the final assessment (β = 1.51, *p* = 0.0101). Other variables, including age, BMI, preoperative rehabilitation, time to rehabilitation, PSA level, presence of EPE, SVI, and ISUP grade, were not significantly associated with continence status at follow-up.

**Figure 3:**
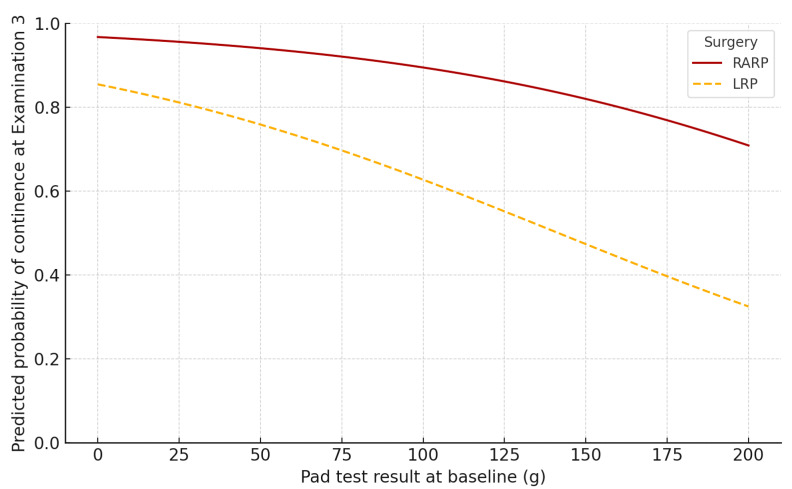
Predicted probability of full continence after Examination 3 by type of surgery and baseline pad test result. Caption: Predicted probabilities of achieving full continence (pad test ≤2 g) at the final assessment are shown as a function of baseline pad test result, stratified by surgical approach: RARP and LRP. Note: The solid line represents RARP; the dashed line represents LRP. Predictions are based on a multivariable logistic regression model adjusted for baseline pad test result, age, BMI, preoperative PSA, preoperative rehabilitation, time to rehabilitation, and pathological features (ISUP grade, EPE, and SVI).

Based on the fitted logistic regression model, we analyzed the predicted probability of achieving full continence (defined as a pad test result ≤2 g) at the third evaluation (Examination 3), as a function of baseline pad test result and type of surgery (Figure 3). The analysis revealed that the likelihood of achieving continence decreased with increasing baseline incontinence severity. However, across all baseline severity levels, patients who underwent RARP demonstrated consistently higher predicted probabilities of reaching continence compared to those treated with LRP. These differences were particularly evident among patients with moderate to severe incontinence at the start of rehabilitation.

#### 3.2.4. Likelihood of Completing Rehabilitation After the Second Visit

An ordinal logistic regression analysis was conducted to identify factors associated with the time required to achieve full continence, defined as a pad test result ≤2 g (Table A2). The dependent variable (Examination stop) indicated the rehabilitation visit after which the patient reached continence (second, third, or later). The results showed that greater baseline incontinence severity, measured by a higher pad test result at Examination 1, was significantly associated with delayed continence recovery (β = 0.01, *p* = 0.0006). Additionally, a longer time to initiation of rehabilitation was significantly associated with slower improvement (β = 0.03, *p* = 0.0348). Other variables, including age, BMI, preoperative PSA level, type of surgery, and histopathological features (ISUP grade, EPE, and SVI), were not significantly associated with the timing of continence recovery.

## 4. Discussion

Our findings demonstrate that pelvic floor muscle physiotherapy is an effective method for managing UI in patients after radical prostatectomy. At six months postoperatively, 80% of patients had achieved continence. The greatest improvements were observed among individuals with less severe incontinence after surgery and those who initiated rehabilitation earlier. Preoperative physiotherapy was independently associated with a greater likelihood of UI stage improvement. Patients treated with RARP had significantly higher odds of achieving continence compared to those who underwent conventional LRP, particularly among those with moderate to severe incontinence. At baseline, the ordinal regression model revealed that the presence of extraprostatic extension (EPE) was associated with higher odds of more severe urinary incontinence, whereas higher ISUP grades (2–4) were associated with lower odds of severe incontinence compared with ISUP 1. These associations should be interpreted cautiously, as they may reflect case-mix characteristics rather than a true biological relationship. For instance, patients with higher ISUP grades might have received more careful perioperative management or earlier physiotherapy referral. Nevertheless, the observed directionality aligns with the multivariable estimates presented in Table A1 and confirms that RARP independently predicted lower baseline UI severity. In Figure 1, the combination of ISUP 2 and the presence of EPE (1) appeared to predict higher probabilities of severe UI stages. However, this subgroup included fewer than ten patients, and the wide confidence intervals indicate that this observation likely reflects random variability rather than a meaningful clinical association.

Our findings regarding the effectiveness of physiotherapy are consistent with previous studies demonstrating the benefits of pelvic floor muscle training after radical prostatectomy [3,12,15,20,21]. In our protocol, patients began formal rehabilitation approximately one month after catheter removal; however, they were encouraged to start pelvic floor exercises independently as early as the first day post-catheter removal. This delay in supervised initiation may have limited the effectiveness of early training, as patients might not have performed the exercises correctly due to postoperative discomfort or uncertainty. Immediate access to physiotherapist-guided training could help prevent such errors by enabling early correction of technique. Another issue is the involuntary and sustained contraction of pelvic floor muscles following surgery, which can occur subconsciously in response to anxiety about leakage or to protect the surgical site [22]. Persistent tension may hinder proper muscle relaxation and coordinated activation. In such cases, timely correction and individualized guidance from a physiotherapist could lead to more effective training and faster continence recovery. However, this approach is feasible primarily for patients who received preoperative rehabilitation, as they are already familiar with the correct technique and can focus on refinement rather than initial learning.

In our study, participation in preoperative rehabilitation was independently associated with a greater likelihood of improvement in UI severity. During preoperative sessions, patients received verbal instructions on how to correctly contract and relax the pelvic floor muscles [23]. Ultrasound imaging was used to assess muscle function and to provide real-time visual feedback to enhance patients’ awareness of proper technique. Additionally, emphasis was placed on avoiding abdominal straining and breath-holding common mistakes that can compromise the effectiveness of pelvic floor exercises. These components are often challenging for patients to master initially, underscoring the importance of training by a specialized physiotherapist who can apply various tools and techniques to ensure correct muscle activation [22,23,24]. Although long-term adherence and consistency are essential for lasting results, the study by Khorrami et al. demonstrated that even a single preoperative physiotherapy session may provide meaningful short-term benefits in UI outcomes following RP [25]. The positive impact of early rehabilitation initiation aligns with previous findings; however, our study is among the few that have accounted for this factor in multivariable analysis. While the value of preoperative physiotherapy remains debated, our data support its role as a preparatory component of prostate cancer treatment. Evidence suggests that patients receiving preoperative training are more likely to regain continence within the first three months, although its benefit appears to diminish by six months postoperatively. We advocate that all surgeons recommend preoperative rehabilitation, as it contributes to improved quality of life [25]. Beyond the physiological benefits, preoperative rehabilitation also offers psychological support. Patients awaiting treatment often experience anxiety related to the uncertainty of their cancer prognosis and concerns about postoperative side effects [26]. Interaction with a physiotherapist and peer patients fosters a sense of care and motivation, empowering patients to actively engage in their recovery process.

Our findings indicate that the baseline pad test result can be used to stratify patients by the severity of UI, enabling a more personalized rehabilitation plan. In this study, we used the 1 h pad test (1h-PT), acknowledging that the 24 h version (24 h-PT) would have provided a more comprehensive assessment of incontinence severity [27,28,29]. However, due to logistical constraints and concerns about patient compliance, we opted for the shorter test, which allowed us to maintain full control over its administration and the measurement process. Importantly, higher baseline pad test results were significantly associated with a lower likelihood of regaining continence. In addition to objective testing, we assessed UI based on patient-reported usage of hygiene materials, consistent with approaches adopted in other studies [30]. Notably, we observed considerable variability in patients’ subjective perception of incontinence: for the same measured urine loss (e.g., 5 g), some patients used four pads per day, while others used only one over 24 h. This discrepancy may reflect individual hygiene preferences or economic considerations, as pad usage likely varies between populations [31]. Furthermore, some patients were classified as fully continent by the pad test yet reported using at least one pad per day, which, by definition, qualifies as incontinence [8,31]. These findings underscore the need for careful interpretation of both objective and subjective measures of UI in clinical and research settings.

These observations are consistent with recent evidence from a multicenter study published in European Urology Surgery [32], which demonstrated that individualized perioperative PFMT significantly improved early continence recovery and reduced time to functional independence. Together with our findings, this supports the growing consensus that structured, personalized rehabilitation protocols should be integrated into standard perioperative management of prostatectomy patients.

Patients who underwent RARP were more likely to achieve continence than those treated with conventional LRP. Among individuals with moderate to severe UI, the probability of achieving continence within six months was notably higher after RARP. These findings are consistent with prior studies suggesting a potential advantage of RARP over LRP in terms of faster recovery of sphincter function. Several reports have shown that patients undergoing RARP experience lower rates of postoperative incontinence compared to those treated with LRP [4,33,34,35]. It should be noted that this apparent advantage may primarily reflect more favorable baseline continence rather than a greater treatment effect. In our analysis, RARP predicted lower initial UI severity but did not significantly influence the magnitude of improvement during rehabilitation. However, one study comparing patient-reported quality of life following different surgical techniques found no statistically significant difference in the use of hygiene products 12 months after surgery [36]. These inconsistent results may stem from variations in surgeon experience and expertise. In our study, all RARP procedures were performed by two surgical teams with comparable levels of experience and similar annual case volumes. Previous research has demonstrated that continence outcomes tend to improve when procedures are performed by high-volume surgeons. Performing more than 50 radical prostatectomies per year has been associated with better continence recovery after surgery [37].

The observed advantage of RARP over LRP should be interpreted with caution. Several perioperative and surgeon-related factors that were not available in our dataset—such as nerve-sparing status, bladder-neck preservation, posterior reconstruction, membranous urethral length, prostate volume, catheterization time, anastomotic leakage, and perioperative complications—may have influenced continence recovery. Moreover, surgeon experience and learning curve are well-known determinants of functional outcomes after radical prostatectomy. Although in our center all RARP procedures were performed by two high-volume teams with comparable case loads, the potential impact of these unmeasured factors cannot be excluded. Therefore, the difference observed between surgical approaches may reflect, at least in part, confounding by case-mix and surgical expertise rather than the robotic technique itself. Overall, our findings do not allow for a causal inference regarding the superiority of one surgical technique over another. Rather, they highlight the multifactorial nature of continence recovery, where both surgical precision and comprehensive postoperative rehabilitation play complementary roles.

### Study Limitations

This study has several limitations. First, we used a 1 h pad test rather than the more comprehensive 24 h version, which could have provided a more accurate assessment of urinary leakage. Second, the study lacked randomization and did not include a control group of patients who did not undergo physiotherapy, making it impossible to isolate the independent effect of the rehabilitation intervention. Third, the follow-up period was relatively short and may not fully capture long-term continence outcomes. Additionally, although the study adjusted for multiple clinical and pathological factors, the influence of potential confounders such as surgeon experience, patient adherence to exercise protocols, or psychosocial factors could not be fully excluded. Finally, while the ordinal and logistic regression models accounted for various predictors, the sample size may have limited the statistical power to detect smaller effects in some subgroups.

Another limitation concerns the lack of detailed data on surgeon experience and surgical technique parameters, such as nerve-sparing or bladder-neck preservation. Although all RARP procedures were performed by two high-volume teams with comparable caseloads, we cannot fully exclude the potential influence of operator-specific factors on continence outcomes.

In addition, we acknowledge limitations related to the assessment of adherence to the rehabilitation program. Although attendance at supervised sessions was systematically recorded, adherence to the home-based exercise component was based on patient self-reports and physiotherapist notes rather than objective monitoring. Therefore, some degree of reporting bias cannot be excluded. Nevertheless, the high attendance rate (>90%) and the consistency between intention-to-treat and per-protocol analyses support the robustness of the main findings.

Nevertheless, the study contributes valuable evidence on the effectiveness of pelvic floor rehabilitation following radical prostatectomy and highlights the importance of early and preoperative interventions in improving continence outcomes.

In summary, our findings suggest that pelvic floor muscle physiotherapy, particularly when implemented both preoperatively and early postoperatively, can substantially accelerate the recovery of urinary continence after radical prostatectomy. Early initiation of rehabilitation, patient education, and individualized therapy plans based on objective indicators should be considered standard components of postoperative care. Future research should focus on assessing the long-term effects of physiotherapy and establishing optimal rehabilitation protocols tailored to specific patient subgroups.

## 5. Conclusions

This study confirms that pelvic floor rehabilitation after radical prostatectomy is an effective intervention for improving urinary continence outcomes. The most substantial gains were observed during the first three rehabilitation sessions, with 80% of patients achieving full continence (pad test ≤ 2 g) by the third evaluation. A higher baseline severity of incontinence and longer delays in initiating therapy were significantly associated with slower or incomplete recovery. In contrast, preoperative physiotherapy and earlier rehabilitation onset independently predicted greater improvement in both objective (pad test) and clinical (UI stage) outcomes. Furthermore, patients who underwent RARP had significantly higher probabilities of achieving continence than those treated with laparoscopic approaches, especially in cases of moderate to severe initial incontinence. These findings underscore the importance of early, individualized pelvic floor training, particularly for patients at elevated risk of prolonged urinary incontinence.

## Figures and Tables

**Table 1:** Baseline characteristics of the study group, overall and stratified by urinary incontinence stage.

Characteristic	Total Group (*n* = 182)	UI Stage 0 (*n* = 64)	UI Stage 1 (*n* = 23)	UI Stage 2 (*n* = 47)	UI Stage 3 (*n* = 48)
Age (years), mean (SD)	66.1 (6.5)	65.5 (6.0)	63.1 (6.9)	66.8 (6.7)	67.7 (6.2)
BMI (kg/m^2^), mean (SD)	28.2 (3.6)	28.0 (3.7)	27.8 (3.4)	29.2 (4.0)	27.8 (3.2)
PSA before surgery (ng/mL), mean (SD)	9.2 (7.8)	8.4 (4.7)	11.3 (11.4)	8.4 (7.4)	10.0 (9.4)
PSA post-surgery (ng/mL), mean (SD)	0.2 (1.5)	0.1 (0.5)	0.2 (0.6)	0.5 (2.8)	0.1 (0.6)
Rehabilitated before surgery, *n* (%)					
No	36 (19.8)	12.0 (18.8)	3.0 (13.0)	6.0 (12.8)	15.0 (31.3)
Yes	146 (80.2)	52.0 (81.3)	20.0 (87.0)	41.0 (87.2)	33.0 (68.8)
Time to rehabilitation (days), mean (SD)	36.1 (14.0)	35.1 (6.0)	38.9 (13.8)	36.7 (21.9)	35.6 (11.8)
Pad test result at Examination 1 (g), mean (SD)	43.9 (68.9)	3.2 (15.7)	4.3 (1.8)	30.1 (12.4)	130.8 (83.1)
Pad test result at Examination 3 (g), mean (SD)	8.0 (22.9)	0.4 (1.4)	0.6 (0.7)	5.8 (16.4)	23.7 (37.3)
Δ Pad test result (g), mean (SD)	36.0 (63.4)	2.9 (15.8)	3.7 (1.9)	24.3 (18.9)	107.1 (86.5)
Type of surgery, *n* (%)					
LRP	106 (58.2)	19.0 (29.7)	16.0 (69.6)	36.0 (76.6)	35.0 (72.9)
RARP	76 (41.8)	45.0 (70.3)	7.0 (30.4)	11.0 (23.4)	13.0 (27.1)
pT, *n* (%)					
pT1	2 (1.1)	na	1.0 (4.4)	na	1.0 (2.1)
pT2	126 (69.2)	53.0 (82.8)	12.0 (52.2)	34.0 (72.3)	27.0 (56.3)
pT3	54 (29.7)	11.0 (17.2)	10.0 (43.5)	13.0 (27.7)	20.0 (41.7)
pN, *n* (%)					
Nx	17 (9.3)	6.0 (9.4)	3.0 (13.0)	4.0 (8.5)	4.0 (8.3)
N0	163 (89.6)	58.0 (90.6)	20.0 (87.0)	42.0 (89.4)	43.0 (89.6)
N1	2 (1.1)	na	na	1.0 (2.1)	1.0 (2.1)
pM, *n* (%)					
M0	161 (88.5)	60.0 (93.8)	18.0 (78.3)	41.0 (87.2)	42.0 (87.5)
M1	4 (2.2)	1.0 (1.6)	2.0 (8.7)	1.0 (2.1)	na
Mx	17 (9.34)	3 (4.7)	3 (13.1)	5 (10.6)	6 (12.5)
GS1, *n* (%)					
GS1 → 3	58 (31.9)	26.0 (40.6)	9.0 (39.1)	8.0 (17.0)	15.0 (31.3)
GS1 → 4	121 (66.5)	38.0 (59.4)	14.0 (60.9)	37.0 (78.7)	32.0 (66.7)
GS1 → 5	3 (1.6)	na	na	2.0 (4.3)	1.0 (2.1)
GS2, *n* (%)					
GS2 → 3	77 (42.3)	22.0 (34.4)	11.0 (47.8)	22.0 (46.8)	22.0 (45.8)
GS2 → 4	89 (48.9)	38.0 (59.4)	11.0 (47.8)	18.0 (38.3)	22.0 (45.8)
GS2 → 5	16 (8.8)	4.0 (6.3)	1.0 (4.4)	7.0 (14.9)	4.0 (8.3)
GS, *n* (%)					
GS → 6	16 (8.8)	4.0 (6.3)	3.0 (13.0)	4.0 (8.5)	5.0 (10.4)
GS → 7	100 (54.9)	38.0 (59.4)	14.0 (60.9)	21.0 (44.7)	27.0 (56.3)
GS → 8	50 (27.5)	20.0 (31.3)	5.0 (21.7)	14.0 (29.8)	11.0 (22.9)
GS → 9	16 (8.8)	2.0 (3.1)	1.0 (4.4)	8.0 (17.0)	5.0 (10.4)
Persistent PSA, *n* (%)					
No	158 (86.8)	56.0 (87.5)	19.0 (82.6)	39.0 (83.0)	44.0 (91.7)
Yes	24 (13.2)	8.0 (12.5)	4.0 (17.4)	8.0 (17.0)	4.0 (8.3)
EPE, *n* (%)					
EPE 0	129 (70.9)	53.0 (82.8)	14.0 (60.9)	33.0 (70.2)	29.0 (60.4)
EPE 1	47 (25.8)	10.0 (15.6)	7.0 (30.4)	12.0 (25.5)	18.0 (37.5)
EPE 2	6 (3.3)	1.0 (1.6)	2.0 (8.7)	2.0 (4.3)	1.0 (2.1)
SVI, *n* (%)					
No	162 (89.0)	60.0 (93.8)	18.0 (78.3)	41.0 (87.2)	43.0 (89.6)
Yes	20 (11.0)	4.0 (6.3)	5.0 (21.7)	6.0 (12.8)	5.0 (10.4)
EAU, *n* (%)					
EAU 1	9 (4.9)	2.0 (3.1)	3.0 (13.0)	2.0 (4.3)	2.0 (4.2)
EAU 2	139 (76.4)	56.0 (87.5)	16.0 (69.6)	36.0 (76.6)	31.0 (64.6)
EAU 3	34 (18.7)	6.0 (9.4)	4.0 (17.4)	9.0 (19.2)	15.0 (31.3)
ISUP, *n* (%)					
ISUP 1	16 (8.8)	4.0 (6.3)	3.0 (13.0)	4.0 (8.5)	5.0 (10.4)
ISUP 2	39 (21.4)	20.0 (31.3)	6.0 (26.1)	3.0 (6.4)	10.0 (20.8)
ISUP 3	61 (33.5)	18.0 (28.1)	8.0 (34.8)	18.0 (38.3)	17.0 (35.4)
ISUP 4	50 (27.5)	20.0 (31.3)	5.0 (21.7)	14.0 (29.8)	11.0 (22.9)
ISUP 5	16 (8.8)	2.0 (3.1)	1.0 (4.4)	8.0 (17.0)	5.0 (10.4)

Abbreviations: BMI, body mass index; PSA, prostate-specific antigen; LRP, laparoscopic radical prostatectomy; RARP, robot-assisted radical prostatectomy; pT, pathological tumor stage; pN, pathological nodal stage; pM, pathological metastatic stage; GS1, primary Gleason pattern; GS2, secondary Gleason pattern; GS, total Gleason score (sum of GS1 and GS2); EPE, extraprostatic extension; SVI, seminal vesicle invasion; EAU, European Association of Urology risk classification; ISUP, International Society of Urological Pathology; SD, standard deviation. Note: Data are presented as mean (SD) for continuous variables and number (percentage) for categorical variables. The 1 h pad test quantifies urinary leakage in grams and was performed at baseline (Examination 1). Δ Pad test result represents the difference in pad test values between baseline and follow-up (Examinations 1 and 3), with higher positive values indicating greater improvement in continence. EAU risk groups: EAU1 (low risk): PSA < 10 ng/mL, ISUP 1, cT1–T2a, EAU2 (intermediate risk): PSA 10–20 ng/mL, ISUP 2–3, cT2b–T2c, EAU3 (high risk): PSA > 20 ng/mL, ISUP ≥ 4, ≥T3. ISUP Grade Groups: ISUP 1: Gleason ≤ 6, ISUP 2: 3 + 4, ISUP 3: 4 + 3, ISUP 4: 8, ISUP 5: 9–10. Arrows indicate transitions between Gleason primary and secondary patterns (e.g., GS1 → 3). ‘na’ indicates data not applicable due to absence of cases in a given category.

**Table 2:** Results of ordinal logistic regression for change in UI stage between Examination 1 and 3.

Characteristic	β (95% CI)	*p*
Age (years)	0.0 (−0.05, 0.05)	0.87
BMI (kg/m^2^)	−0.01 (−0.1, 0.09)	0.8582
Rehabilitation before surgery (Yes vs. No)	−1.27 (−2.17, −0.36)	0.0061
Time to rehabilitation (days)	−0.04 (−0.06, −0.01)	0.0026
Type of surgery (RARP vs. LRP)	0.31 (−0.51, 1.14)	0.4564
PSA before surgery (ng/mL)	−0.01 (−0.05, 0.02)	0.5235
EPE 1 vs. EPE 0	−0.36 (−1.22, 0.51)	0.4181
EPE 2 vs. EPE 0	−1.16 (−3.15, 0.82)	0.2511
SVI (Yes vs. No)	−0.17 (−1.47, 1.14)	0.8012
ISUP 2 vs. ISUP 1	0.28 (−1.12, 1.67)	0.698
ISUP 3 vs. ISUP 1	−0.12 (−1.41, 1.16)	0.8494
ISUP 4 vs. ISUP 1	−0.46 (−1.83, 0.91)	0.511
ISU P5 vs. ISUP 1	0.64 (−1.01, 2.29)	0.4466

Note: Estimates are presented as regression coefficients (β), 95% confidence intervals (CI), and *p*-values. Negative β values indicate a greater likelihood of improvement in UI stage. Categorical variables were dummy-coded relative to reference categories.

**Table 3:** Logistic regression model predicting full continence (pad test ≤2 g) after rehabilitation (Examination 3).

Characteristic	β (95% CI)	*p*
Pad test result at baseline (g)	−0.01 (−0.02, −0.01)	0.0001
Age (years)	−0.05 (−0.12, 0.02)	0.1646
BMI (kg/m^2^)	−0.03 (−0.15, 0.1)	0.6887
Rehabilitation before surgery (Yes vs. No)	−0.58 (−1.72, 0.57)	0.3229
Time to rehabilitation (days)	0.0 (−0.01, 0.02)	0.5766
Type of surgery (RARP vs. LRP)	1.09 (0.02, 2.17)	0.0468
PSA before surgery (ng/mL)	−0.02 (−0.07, 0.03)	0.4403
EPE 1 vs. EPE 0	−0.86 (−1.87, 0.16)	0.0971
EPE 2 vs. EPE 0	−0.11 (−2.95, 2.73)	0.9397
SVI (Yes vs. No)	−0.19 (−1.68, 1.31)	0.8058
ISUP 2 vs. ISUP 1	0.75 (−1.06, 2.56)	0.4152
ISUP 3 vs. ISUP 1	1.07 (−0.68, 2.81)	0.2302
ISUP 4 vs. ISUP 1	0.62 (−1.23, 2.46)	0.5107
ISUP 5 vs. ISUP 1	1.62 (−0.68, 3.92)	0.1675

Note: Estimates are presented as regression coefficients (β), 95% confidence intervals (CI), and *p*-values. A negative β value indicates reduced odds, and a positive β value indicates increased odds of achieving full continence. Categorical variables were coded relative to their respective reference categories. Baseline pad test result refers to the measurement at Examination 1.

## Data Availability

The data supporting the findings of this study are not publicly available due to privacy and ethical restrictions. Reasonable requests for access to anonymized data may be considered by the corresponding author.

## Appendix A

**Table A1:** Results of ordinal (logistic) regression model with UI stage at baseline as the dependent variable.

Characteristic	β (95% CI)	*p*
Age (years)	0.03 (−0.01, 0.08)	0.1376
BMI (kg/m^2^)	−0.02 (−0.1, 0.06)	0.5775
Rehabilitation before surgery (Yes vs. No)	−0.69 (−1.43, 0.06)	0.0708
Time to rehabilitation (days)	0.01 (−0.01, 0.03)	0.4349
Type of surgery (RARP vs. LRP)	−1.64 (−2.29, −1.0)	<0.001
PSA before surgery (ng/mL)	0.01 (−0.02, 0.05)	0.5066
EPE1 vs. EPE 0	0.87 (0.1, 1.63)	0.0266
EPE2 vs. EPE 0	0.20 (−1.57, 1.96)	0.8282
SVI (Yes vs. No)	−0.55 (−1.69, 0.58)	0.3386
ISUP 2 vs. ISUP 1	−1.43 (−2.57, −0.3)	0.0135
ISUP 3 vs. ISUP 1	−1.07 (−2.15, 0.01)	0.0516
ISUP 4 vs. ISUP 1	−1.58 (−2.73, −0.42)	0.0076
ISUP 5 vs. ISUP 1	−0.43 (−1.88, 1.02)	0.5605

Note: The model included all analyzed clinical and demographic variables. Estimates are presented as regression coefficients (β), 95% confidence intervals (CI), and *p*-values. Negative β values indicate a decreased likelihood of being classified into a higher UI stage. Categorical variables were coded as binary indicators relative to the reference category.

**Table A2:** Ordinal logistic regression model assessing factors influencing the time to achieve full continence (pad test result ≤ 2 g).

Characteristic	β (95% CI)	*p*
Pad test result at baseline (g)	0.01 (0.00, 0.01)	0.0006
Age (years)	0.04 (−0.01, 0.1)	0.1325
BMI (kg/m^2^)	0.04 (−0.06, 0.14)	0.4341
Time to rehabilitation (days)	0.03 (0.00, 0.05)	0.0348
PSA before surgery (ng/mL)	−0.0 (−0.05, 0.04)	0.8366
Rehabilitation before surgery (Yes vs. No)	0.22 (−0.68, 1.12)	0.6314
Type of surgery (RARP vs. LRP)	−0.7 (−1.59, 0.18)	0.1192
EPE 1 vs. EPE 0	0.52 (−0.4, 1.45)	0.2664
EPE 2 vs. EPE 0	−0.02 (−2.08, 2.03)	0.9834
SVI (Yes vs. No)	0.12 (−1.27, 1.5)	0.871
ISUP 2 vs. ISUP 1	−1.78 (−3.29, −0.27)	0.021
ISUP 3 vs. ISUP 1	−0.73 (−2.09, 0.62)	0.29
ISUP 4 vs. ISUP 1	−0.27 (−1.74, 1.2)	0.722
ISUP 5 vs. ISUP 1	−1.0 (−2.72, 0.71)	0.2522

Note: Ordinal logistic regression model assessing the association between clinical and pathological characteristics and the time to achieving full continence, defined as a pad test result ≤ 2 g. The dependent variable (Examination stop) indicates the rehabilitation visit at which continence was achieved (2, 3, or >3). Estimates are presented as regression coefficients (β), 95% confidence intervals (CI), and *p*-values. Categorical variables were dummy-coded relative to reference categories. Pad test result refers to the baseline value (Examination 1).
